# Quantum Dot-Based Dual-Fluorescence Aptasensing Platform Using Interface-Engineered MXene for Multiplex Protein Detection

**DOI:** 10.3390/s26123856

**Published:** 2026-06-17

**Authors:** Qichen Yang, Chun Yang, Mingzhu Liu, Nan Su, Jingran Sun, Jian Hou, Yixue Fu, Jin Wu, Yu Wang, Yuan Peng, Jialei Bai, Ying Liu, Zunquan Zhao

**Affiliations:** 1 School of Public Health, Inner Mongolia Medical University, Hohhot 010059, China; yangqichen0010@163.com (Q.Y.); m18851110336@163.com (C.Y.); 2 Tianjin Key Laboratory of Risk Assessment and Control Technology for Environment and Food Safety, Military Medical Sciences Academy, Academy of Military Sciences, Tianjin 300050, China; lmzazhu@163.com (M.L.); sunan2009@126.com (N.S.); sunjr@163.com (J.S.); 18722096749@163.com (J.H.); yzzsbky@126.com (Y.F.); wujinlch@163.com (J.W.); wangyuyu9210@163.com (Y.W.); dalidao@139.com (Y.P.); baijialeitj@163.com (J.B.)

**Keywords:** MXene, quantum dot, aptasensor, simultaneous detection, click chemistry

## Abstract

**Highlights:**

**What are the main findings?**

- A dual-color fluorescence aptasensing system utilizing quantum dots and polydopamine-coated MXene was developed for simultaneous detection of spike and hemagglutinin proteins.
- Centrifugation-assisted signal separation, modular storage of aptamer lyophilized powder, and copper-free click chemistry-mediated self-assembly enabled sensitive multiplex detection.

**What are the implications of the main findings?**

- The system offers a modular strategy that can be adapted to other respiratory pathogen targets by simply exchanging the aptamer lyophilized powder.
- Validation in artificial saliva suggests potential practicality for point-of-care application in clinical and public health settings.

**Abstract:**

Antigen detection provides rapid and convenient diagnosis of respiratory infections. This study develops an innovative dual-fluorescence aptasensing method based on polydopamine-functionalized MXene (PDA-MXene) for the simultaneous detection of spike protein and hemagglutinin protein. The method employs green- and red-emitting quantum dot (QD) probes as fluorescence reporters, and the PDA-MXene as an effective adsorption and separation substrate. Coupled with a centrifugation-assisted separation strategy, this design method reduces background interference and enhances detection reliability. The method demonstrates good analytical performance, with detection limits of 0.82 ng/mL for spike protein and 2.11 ng/mL for hemagglutinin protein in single-channel mode. The dual-channel mode enables reliable and simultaneous quantification of both target proteins with minimal spectral cross-talk. Furthermore, this method exhibits high specificity against interferents including ions, proteins, and toxins. Artificial saliva, chosen as real sample, is spiked with target proteins to investigate the practical applicability of the method, showing recovery rates for both target proteins between 100 and 114 sensing strategy is simple to operate and allows the detection of new targets by simply replacing the azide-modified aptamer lyophilized powder. It therefore holds promising application for the simultaneous detection of multiple proteins in point-of-care testing and health monitoring fields.

## 1. Introduction

Respiratory infections pose an ongoing threat to public health systems worldwide [1]. Effectively combating respiratory infection epidemics requires diagnostic tools that can be deployed at low cost in hospitals, community clinics, high-traffic public spaces, and even in the home [2]. An ideal point-of-care (POC) diagnostic tool should combine reliable, rapid testing capability with low cost, environmental stability, and the ability to be stockpiled on a large scale. Due to their speed and simplicity, antigen tests better meet these requirements compared to laboratory-dependent nucleic acid amplification detection techniques [3,4]. For instance, the Severe Acute Respiratory Syndrome Coronavirus 2 (SARS-CoV-2) spike protein is a key diagnostic target due to its high immunogenicity and direct association with viral particle infectivity [5]. It also serves as an important biomarker for post-acute sequelae of COVID-19 (PASC), with studies showing that the protein remains detectable at pg level in infected patients for up to one year after infection [6,7]. Moreover, the hemagglutinin (HA) protein is a key target for diagnosing active influenza A virus infections and tracking viral transmission [8,9,10].

In recent years, POC antigen detection methods for respiratory pathogens have been developed [11,12,13,14]. A multiplexed capillary-flow driven immunoassay was reported for simultaneous detection of H1N1 HA protein and SARS-CoV-2 nucleocapsid protein, with detection limits (LOD) of 840 pg/mL for HA and 133 pg/mL protein, respectively [12]. However, most antigen detection methods rely on antibodies, which increases their cost and thus limits their widespread adoption by consumers [15]. A peptide-based electrochemical biosensor was developed for SARS-CoV-2 spike protein detection, achieving a LOD of 18.2 ng/mL [16]. The method was validated on clinical nasopharyngeal swab samples, distinguishing patients diagnosed with COVID-19 and healthy individuals and differentiating viral loads. Antigen detection using aptasensor is also a promising alternative to antibody-based antigen detection [17,18]. Aptamers have advantages such as low cost, good thermal stability, and ease of chemical modification, making them well-suited for long-term storage and large-scale production [19]. However, most aptasensors require complex sensing or signal enhancement designs to achieve high sensitivity [17,20,21]. Although aptamers stored as lyophilized powders have a long shelf life, their long-term functional stability may decline significantly once they are immobilized on a solid surface or conjugated with nanotags [22]. This loss of stability in the assembled state is a major obstacle to the commercialization of aptasensor-based POC diagnostic tools.

Aptasensors employing two-dimensional nanoquenchers offer a promising approach for low-cost and effective antigen detection [23,24]. MXenes possess excellent conductivity, high surface area, and versatile surface chemistry, making them suitable for the construction of turn-on biosensors [25]. In such systems, fluorescently labeled aptamers adsorb onto the MXene surface and undergo fluorescence quenching; recognition between the target and the aptamer disrupts the adsorption interaction between the aptamer and the MXene, and the dissociation of the aptamer probe results in measurable fluorescence recovery [23,25]. The use of ultrathin MXene nanosheets enhances interfacial contact with the aptamer, thereby improving target accessibility and signal transduction efficiency [26]. Nevertheless, the oxidative instability of MXenes in aqueous environments remains a key challenge, threatening the long-term storage stability and practical deployment of such aptasensors [27]. Interface modification strategies, such as polydopamine coating, offer effective approaches to improve the water stability and antioxidant stability of MXene nanosheets [28]. Furthermore, the sensitivity of such aptasensors depends directly on the intensity of the signal label on the aptamer. Nano-labeling offers a powerful method for enhancing this signal [29], but the resulting aptamer probes are often limited in terms of storage conditions and long-term stability, as most nanolabels cannot be stored in frozen conditions [30]. Modular self-assembly methods, such as click chemistry, may enable rapid, on-demand labeling while maintaining aptamer stability [31,32,33], offering a promising path for the development of storable aptamer-based diagnostic tools.

This study developed a polydopamine-coated MXene aptasensor for simultaneous detection of two major respiratory viral antigens: the spike (S1) protein of SARS-CoV-2 virus, and the hemagglutinin (HA) protein of Influenza A virus (H1N1 subtype). This aptasensor combined the quantum dot-labeled aptamers as the recognition elements with the MXene nannosheets as a carrier of quantum dot-labeled aptamer probes. The MXene was coated with polydopamine to enhance its stability. In the detection process, the introduction of a centrifugation separation step enhanced the signal-to-noise ratio, enabling highly sensitive detection.

## 2. Materials and Methods

### 2.1. Reagents and Materials

Amino-functionalized CdSe/ZnS quantum dots were purchased from Wuhan JIAYUAN QUANTUM DOTS Co., Ltd. (Wuhan, China). DBCO-PEG4-NHS ester and lithium fluoride (LiF) were obtained from Aladdin Biochemical Technology Co., Ltd. (Shanghai, China). Titanium aluminum carbide (Ti_3_AlC_2_, 400 mesh) was purchased from XINXI TECHNOLOGY Co., Ltd. (Foshan, China). SARS-CoV-2 spike S1 protein was obtained from Sangon Biotech (Shanghai, China), and H1N1 influenza virus HA protein was purchased from Shandong LANDU BIOLOGY Co., Ltd. (Binzhou, China). The azide-modified aptamers (Apt) specific to S1 protein [34] and HA protein [35] were synthesized and purified using high-performance liquid chromatography (HPLC) by Sangon Biotech (Shanghai, China). The detailed nucleotide sequences are provided in Table S1 in the Supplementary Material. Phosphate-buffered saline (PBS) and dimethyl sulfoxide (DMSO) were purchased from Servicebio (Wuhan, China). Hydrochloric acid (HCl) was acquired from Sinopharm Reagent Co., Ltd. (Shanghai, China). Dopamine hydrochloride was purchased from Alfa Aesar (Shanghai, China). NAP-5 desalting columns (50 preps) were purchased from Cytiva (Shanghai, China). Ultrapure water (18.2 MΩ·cm, Milli-Q) was used throughout the experiments.

### 2.2. Apparatus

The morphology of the synthesized MXene nanomaterials were investigated by scanning electron microscopy (SEM) using a Regulus 8100 microscope (Hitachi, Tokyo, Japan). Fluorescence emission spectra were recorded on a FS5C v2 fluorescence spectrophotometer (Edinburgh Instrument, Livingston, UK). Zeta potential measurements were performed on a Zetasizer Advance Pro system (Malvern Panalytical, Malvern, UK). Fluorescence intensity measurements were performed with a SPARK multimode microplate reader (TECAN, Grödig, Austria).

### 2.3. Synthesis of Polydopamine-Functionalized MXene Nanosheets

Monolayer MXene was synthesized via an acid etching and thermal-ultrasonic delamination method [36,37]. Briefly, 3.2 g of lithium fluoride (LiF, Aladdin, Shanghai, China) was first dissolved in 55 mL of 12 M hydrochloric acid (HCl) within a polytetrafluoroethylene reactor under continuous stirring. Subsequently, 2.0 g of Ti_3_AlC_2_ MAX phase powder (400 mesh) was gradually added into the mixture. The mixture was stirred continuously at room temperature for 24 h (h) to allow the etching reaction to proceed. Subsequently, the resulting mixture was repeatedly washed with Milli-Q water by centrifugation until the pH of the supernatant reached approximately 6, ultimately yielding a multilayer MXene precipitate. The obtained sediment was re-dispersed in Milli-Q water to form a concentrated colloidal dispersion. This dispersion was transferred into a 50 mL centrifuge tube, purged with nitrogen gas to remove oxygen, and sealed. The sealed tube was placed in a water bath at 70 °C and subjected to ultrasonic delamination for 1 h. After ultrasonic treatment, the dispersion was centrifuged at 3500 rpm for 30 min (min). The precipitate was discarded, and the supernatant was collected as the monolayer MXene material.

The surface functionalization of monolayer MXene was achieved via the self-polymerization of dopamine [28]. First, 5.0 mg of dopamine hydrochloride was added to 50 mL of Tris-HCl (50 mM, pH = 10) buffer and stirred for 3 h. Subsequently, under vigorous stirring, the supernatant from the monolayer MXene prepared above was added dropwise to the pre-reacted dopamine solution. The reaction was allowed to proceed for 22 h under continuous stirring to ensure that the dopamine polymerized thoroughly on the surface of the MXene nanosheets. Finally, the product was collected and purified through multiple centrifugations and washes with Milli-Q water to remove unreacted monomers, PDA oligomers, and buffer salts. The purified product was pre-frozen at −80 °C for 12 h and then freeze-dried to obtain polydopamine-functionalized MXene powder, denoted as PDA-MXene.

### 2.4. Preparation of Dual-Color QD@Apt Probes via Copper-Free Click Chemistry

The QD@Apt probes (quantum dot–aptamer conjugates) were synthesized through a copper-free click chemistry conjugation strategy. First, a stock solution of the crosslinker was prepared by dissolving DBCO-PEG4-NHS ester in anhydrous DMSO to 36 mmol/L (mM). Subsequently, 50 μL of 8 μM aqueous suspensions of either red-emitting amino-QD (RQD, Em = 625 nm) or green-emitting amino-QD (GQD, Em = 525 nm) were separately reacted with 50 µL of the DBCO-PEG4-NHS ester solution (36 mM) in 400 µL of 1× PBS (pH = 7.4) at room temperature for 3 h. The crude mixtures were then purified using a NAP-5 desalting column (pre-equilibrated with PBS) to remove excess reagents, yielding purified DBCO-functionalized QD solutions (DBCO-RQD and DBCO-GQD) at approximately 320 nM. The DBCO-QD solutions were stored at 4 °C in the dark. When in use, 3.2 µL of 100 µM azide-modified aptamer (N_3_-S1-Apt or N_3_-HA-Apt) was added into 1 mL of the DBCO-QD solution (1:1 molar ratio) and incubated at room temperature for 1 h. The reaction products are target-specific fluorescent probes, named RQD@Apt and GQD@Apt, respectively. To maintain the activity of the aptamer component under storage conditions consistent with those of other detection elements (PDA-MXene and quantum dots), it is stored at 4 °C in the form of a lyophilized powder of azide-modified aptamers. Upon detection, the lyophilized aptamer powder is dissolved and mixed with the DBCO-QD solution to rapidly assemble the QD@Apt probe via the click chemistry reaction described above. This “store separately, assemble on demand” strategy avoids the loss of binding activity that typically occurs when QD@Apt probes are stored at 4 °C for extended periods.

### 2.5. Determination of S1 Protein in Single-Target Mode

Briefly, 10 μL of PDA-MXene (10 mg/mL) was mixed with 12.5 μL of RQD@S1-Apt probe or GQD@S1-Apt probe (320 nM) and 20 μL of 10× Tris-HCl buffer (0.2 M Tris, 0.992 M NaCl, 0.05 M MgCl_2_·6H_2_O, 0.01 M CaCl_2_·2H_2_O, 0.05 M KCl, pH 8.0) in 157.5 μL of Milli-Q water. The mixture was incubated at room temperature in the dark for 30 min, then centrifuged at 6000 rpm for 3 min to remove the supernatant. The precipitate was resuspended in 200 μL of 1× Tris-HCl buffer, and 10 μL of S1 protein samples at various concentrations (0–100 ng/mL) was added. After reacting at room temperature for 30 min, the mixture was centrifuged at 6000 rpm for 5 min, and the supernatant was collected for fluorescence measurement under 365 nm excitation. In the specificity assay, several potential interferents were tested, including ions (K^+^, Na^+^), bovine serum albumin (BSA), cholesterol (CHOL), small-molecular toxins aflatoxin B1 (AFB1), deoxynivalenol (DON), and HA protein.

### 2.6. Determination of HA Protein in Single-Target Mode

Briefly, 10 μL of PDA-MXene (10 mg/mL) was mixed with 12.5 μL of RQD@HA-Apt probe (320 nM) and 20 μL of 10× Tris-HCl buffer (0.2 M Tris, 0.992 M NaCl, 0.05 M MgCl_2_·6H_2_O, 0.01 M CaCl_2_·2H_2_O, 0.05 M KCl, pH 8.0) in 157.5 μL of ultrapure water. The mixture was incubated at room temperature in the dark for 30 min, then centrifuged at 6000 rpm for 3 min to remove the supernatant. The precipitate was resuspended in 200 μL of 1× Tris-HCl buffer, and 10 μL of HA protein samples at various concentrations (0–100 ng/mL) was added. After reacting at room temperature for 30 min, the mixture was centrifuged at 6000 rpm for 5 min, and the supernatant was collected for fluorescence measurement under 365 nm excitation. In the specificity experiment, several potential interferents were tested, including K^+^, Na^+^, BSA, CHOL, AFB1, DON, and S1 protein.

### 2.7. Simultaneous Determination of S1 Protein and HA Protein in Dual-Target Mode

Briefly, 10 μL of PDA-MXene (20 mg/mL) was mixed with 12.5 μL of GQD@S1-Apt probe (320 nM), 12.5 μL of RQD@HA-Apt probe (320 nM) and 20 μL of 10× Tris-HCl buffer (0.2 M Tris, 0.992 M NaCl, 0.05 M MgCl_2_·6H_2_O, 0.01 M CaCl_2_·2H_2_O, 0.05 M KCl, pH 8.0) in 157.5 μL of ultrapure water. The mixture was incubated in the dark at room temperature for 30 min, followed by centrifugation at 6000 rpm for 3 min to remove the supernatant. The pellet was resuspended in 200 μL of 1× Tris-HCl buffer, and 10 μL of S1 protein or HA protein at varying concentrations (0–100 ng/mL) was added. After reacting at room temperature for 30 min, the mixture was centrifuged at 6000 rpm for 5 min, and the supernatant was collected for fluorescence measurement under 365 nm excitation. The emission intensity of GQD@S1-Apt at 525 nm and RQD@HA-Apt at 625 nm were used to determine the concentrations of S1 protein and HA protein, respectively.

## 3. Results

### 3.1. Design and Working Principle of the Dual-Target Sensing Platform

**Figure 1:**
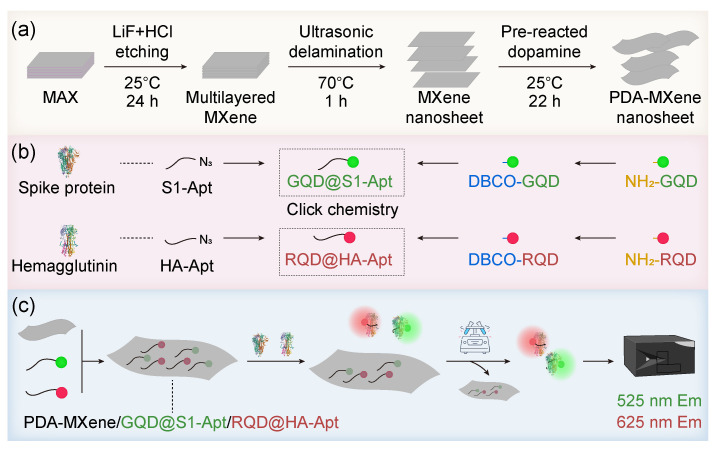
Schematic diagram. (**a**) Illustration of the preparation of PDA-MXene nanosheets. (**b**) Illustration of the preparation of GQD@S1-Apt and RQD@HA-Apt. (**c**) Schematic drawing of the working principle of PDA-MXene/GQD@S1-Apt/RQD@HA-Apt for dual-target simultaneous detection.

As illustrated in Figure 1a, the dual-target sensing platform was constructed starting by preparing ultrathin MXene nanosheets via a simplified thermal-ultrasonic intercalation method [36]. This method effectively delaminates multilayer MXene into thin nanosheets with larger specific surface area. Subsequently, under mild alkaline conditions, pre-reacted polydopamine (PDA) was uniformly polymerized onto the surfaces of these MXene nanosheets, yielding PDA-MXene composites with improved aqueous stability and surface functionality. Figure 1b shows the preparation of the green-emitting quantum dot (GQD) probes and red-emitting quantum dot (RQD) probes. GQD and RQD probes were coupled with S1-specific and HA-specific aptamers, respectively, via a copper-free click chemistry method, yielding GQD@S1-Apt and RQD@HA-Apt probes that retain high affinity while exhibiting strong fluorescence.

Figure 1c presents the working mechanism of the integrated PDA-MXene/GQD@S1-Apt/RQD@HA-Apt sensing system. Initially, both GQD@S1-Apt and RQD@HA-Apt probes are adsorbed onto the PDA-MXene surface through π–π stacking, hydrogen bonding [38,39]. In the absence of target proteins, the system maintains a quenched “signal-off” state through energy or charge transfer mechanisms [23,25]. Upon addition of a sample containing S1 and/or HA proteins, specific aptamer–target binding induces conformational changes in the corresponding QD@Apt probes, weakening their interaction with PDA-MXene and promoting their dissociation from the surface. Therefore, the fluorescence of the corresponding QD is recovered (“signal-on”). The distinct emission wavelengths of GQD (approximately 525 nm) and RQD (approximately 625 nm) enable simultaneous detection of S1 and HA proteins in a single assay, while the centrifugation-assisted separation step further improves sensitivity by removing unbound probes and reducing background interference. The design combines selective recognition and optical multiplexing into a robust and modular platform suitable for multi-target analysis.

### 3.2. Material Synthesis and Characterization

**Figure 2:**
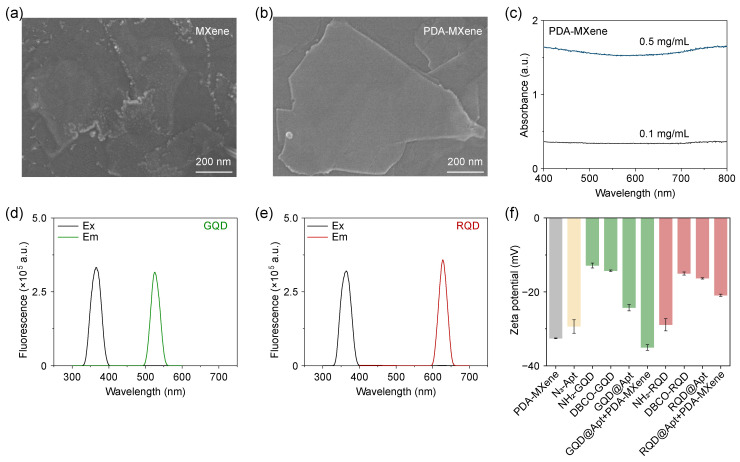
Material synthesis and probe characterization. (**a**) SEM image of pristine MXene nanosheets. (**b**) SEM image of polydopamine-coated PDA-MXene. (**c**) UV-Vis absorption spectrum of PDA-MXene. (**d**) Fluorescence emission spectra of GQD. (**e**) Fluorescence emission spectra of RQD. (**f**) Zeta potential measurements.

Scanning electron microscopy (SEM) analysis was conducted to characterize the successful synthesis of MXene and PDA-MXene nanosheets. The pristine MXene nanosheets exhibited morphological features of oxidation and water erosion at their edges (Figure 2a). In contrast, the edges of the PDA-MXene nanosheets were smoother and more defined, indicating that the polydopamine functionalization effectively prevented degradation of the MXene material (Figure 2b). Ultraviolet–visible (UV-Vis) characterization revealed that MXene (Figure S1) and PDA-MXene and (Figure 2c) possessed a broad absorption spectrum covering 400–800 nm, with a characteristic broad absorption peak in the near-infrared region. In contrast, the UV-Vis absorption intensities of dopamine and pre-reacted PDA were significantly lower (Figures S2 and S3). The GQD and RQD exhibited narrow, well-defined emission peaks at approximately 525 nm and 625 nm, respectively (Figure 2d,e). The successful binding of these quantum dots to S1 protein-specific aptamers via copper-free click chemistry was validated by zeta potential measurements. Significant zeta-potential shifts were observed upon the conjugation (Figure 2f), confirming the formation of quantum dot–aptamer conjugate (QD@Apt probes) probes. Compared with GQD, RQD exhibits a smaller change in zeta potential after conjugation with the aptamer. This may be attributed to differences between the two types of original quantum dots in terms of surface functional group density or particle size. As shown in Figures S4 and S5, the fluorescence intensity of both RQD@Apt and GQD@Apt probes decreased significantly upon the addition of PDA-MXene. These observations support an adsorption-quenching mechanism, whereby PDA-MXene adsorbs the aptamer probes and quenches the fluorescence of the quantum dots carried by the aptamer [25]. However, the fluorescence was not completely turned off, and incomplete quenching was significant.

### 3.3. Optimization of the Operational Workflow for Enhanced Signal-to-Noise Ratio

**Figure 3:**
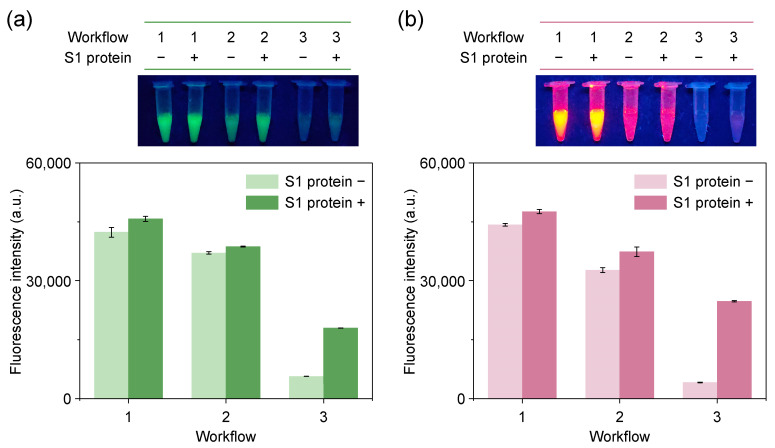
Optimization of the centrifugation-assisted workflows and comparative performance of different QD-aptamer systems. (**a**) Fluorescence responses and fluorescence images of the PDA-MXene/GQD@Apt system under UV illumination obtained from three different workflows. (**b**) Fluorescence responses and fluorescence images of the PDA-MXene/RQDs@Apt under UV illumination obtained from three different workflows. The concentration of S1 protein used for the positive samples in all workflows was 100 ng/mL. Error bars represent standard deviations of triplicate measurements.

The residual fluorescence from QD@Apt probes adsorbed onto PDA-MXene (PDA-MXene/QD@Apt complex) reduces the signal-to-noise ratio of the detection. Therefore, centrifugation purification and signal separation steps were introduced, and three workflows were compared (Figure 3). Workflow 1 represents the conventional method, which involves adding the target (100 ng/mL) directly to a pre-incubated PDA-MXene/QD@Apt mixture, followed by fluorescence measurement of the entire mixture after a 30 min reaction. Workflow 2 introduces a centrifugation purification step prior to target addition. It involves centrifuging the PDA-MXene/QD@Apt mixture (6000 rpm, 3 min) to remove those quantum dots not adsorbed on PDA-MXene, resuspending the mixture to its original volume, and then proceeding with target addition and subsequent fluorescence measurement. Workflow 3 further incorporates a post-reaction signal separation step. Following the same procedure as Workflow 2, the mixture is centrifuged again after the reaction of target recognition, and the fluorescence of the supernatant is measured.

As shown in Figure 3a, for green GQD@Apt, the fluorescence intensity measured under Workflow 3 exhibited the most significant increase upon the addition of the target and had the lowest background signal among the three workflows. Similarly, Figure 3b shows that under Workflow 3, the red GQD@Apt exhibited the strongest target-induced fluorescence response and demonstrated the most pronounced difference compared to the non-target control group. These results support that the centrifugation purification step in Workflow 2 effectively removes quantum dots that have not adsorbed onto PDA-MXene (such as those that have not successfully bound to the aptamer), while the centrifugation step in Workflow 3 effectively removes the intrinsic fluorescence background of PDA-MXene/QD@Apt, thereby enabling the specific detection of probe fluorescence induced by the release from PDA-MXene upon target recognition. In Workflows 2 and 3, the RQD@Apt system demonstrated a stronger fluorescence response than the GQD@Apt system. As shown in the corresponding sample images in Figure 3, the RQD@Apt system also exhibited a visual color change under UV light after the addition of the target, suggesting its potential for semi-quantitative visual detection in scenarios requiring rapid judgment.

### 3.4. Analytical Performance of the Single-Target Detection Method

**Figure 4:**
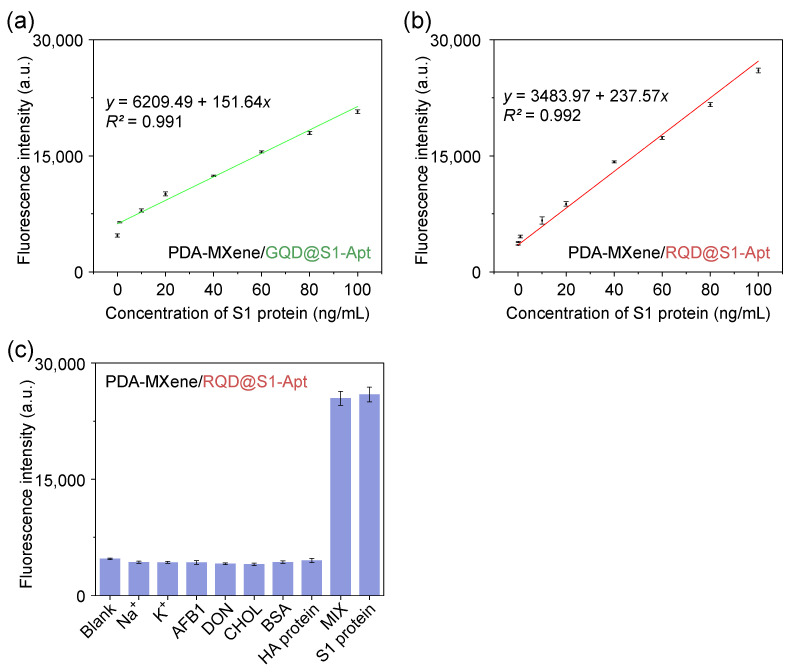
Analytical performance for single-target detection of S1 protein. (**a**) Fluorescence response of the PDA-MXene/GQD@S1-Apt system to varying concentrations of S1 protein (0–100 ng/mL). (**b**) Fluorescence response of the PDA-MXene/RQD@S1-Apt system. Inset: linear calibration curves derived from the data in (**a**,**b**). (**c**) Specificity test of the PDA-MXene/RQD@S1-Apt system against potential interferents. The concentration of S1 protein was 100 ng/mL, while all interferents were tested at 30 μg/mL. Error bars represent triplicate measurements.

Using the optimized Workflow 3, the analytical performance of the fluorescence aptasensor was systematically evaluated. The detection mechanism relies on target-induced conformational changes in the aptamer probe, which releases it from the PDA-MXene carrier and restores fluorescence in the supernatant after centrifugation. The PDA-MXene/GQD@Apt and PDA-MXene/RQD@Apt systems were first assessed for the detection of S1 protein across a concentration range of 0–100 ng/mL. As shown in Figure 4a,b, both systems exhibited a concentration-dependent increase in supernatant fluorescence, with the PDA-MXene/RQD@Apt system demonstrating consistently higher signal intensity. The linear regression equations are y = 6209.49 + 151.64x, R^2^ = 0.991 (PDA-MXene/GQD@Apt system) and y = 3483.97 + 237.57x, R^2^ = 0.992 (PDA-MXene/RQD@Apt system). The sensitivity of PDA-MXene/RQD@Apt system for S1 protein was quantified through calculated detection limits, with a LOD value (3σ/S) of 0.82 ng/mL and corresponding LOQ value (10σ/S) of 2.86 ng/mL. The PDA-MXene/RQD@Apt system was also selected for specificity testing against different interferents. As shown in Figure 4c, a significant fluorescence response phenomenon was observed only in the presence of the S1 protein, suggesting the high specificity of this method.

**Figure 5:**
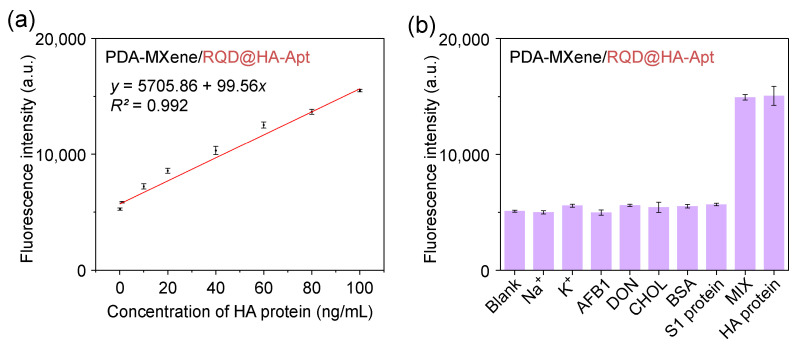
Analytical performance for single-target detection of HA protein. (**a**) Fluorescence response of the PDA-MXene/RQD@HA-Apt system to HA protein concentrations (0.1–100 ng/mL). Inset: linear calibration for HA detection. (**b**) Specificity test of the PDA-MXene/RQD@HA-Apt system. The concentration of HA protein was 100 ng/mL, while all interferents were tested at 30 μg/mL. Error bars represent triplicate measurements.

To verify the scalability of this method, the aptamer was replaced while keeping all other experimental conditions constant. The modified PDA-MXene/RQD@Apt system for HA protein exhibited similar performance (Figure 5a), with a linear calibration curve of y = 5705.86 + 99.56x (R^2^ = 0.992), across the concentration range of 0.1–100 ng/mL. Specificity assessment against the interferent panel again demonstrated exclusive response to the HA target (Figure 5b). The LOD value and LOQ value for HA protein detection were calculated to be 2.11 ng/mL and 7.02 ng/mL, respectively. These results demonstrate that the developed method enables reliable single-target detection with high specificity and sensitivity. The platform also exhibits exceptional versatility, as it can be adapted to different analytes simply by replacing the aptamer lyophilized powder.

### 3.5. Evaluation of Detection Performance in Simulated Real Samples

We further evaluated the PDA-MXene/RQD@Apt system in spike–recovery assays for detecting S1 protein and HA protein, respectively. S1 and HA proteins were added to artificial saliva to prepare spiked samples at concentrations of 0, 10, 20 ng/mL, which were then analyzed. As shown in Table 1, the recovery rates for the S1 protein detection were 100.98–113.05%, and the relative standard deviations (RSD) were 4.10–4.58%. For HA protein detection, the recovery rates were 101.98–105.32%, and the RSDs were 8.67–9.33% (Table 2). These results validate the feasibility of using the PDA-MXene/RQD@Apt platform to detect viral antigens in simulated physiological matrices, suggesting that this platform has potential for practical diagnostic applications.

### 3.6. Development and Evaluation of a Dual-Target Detection System

**Figure 6:**
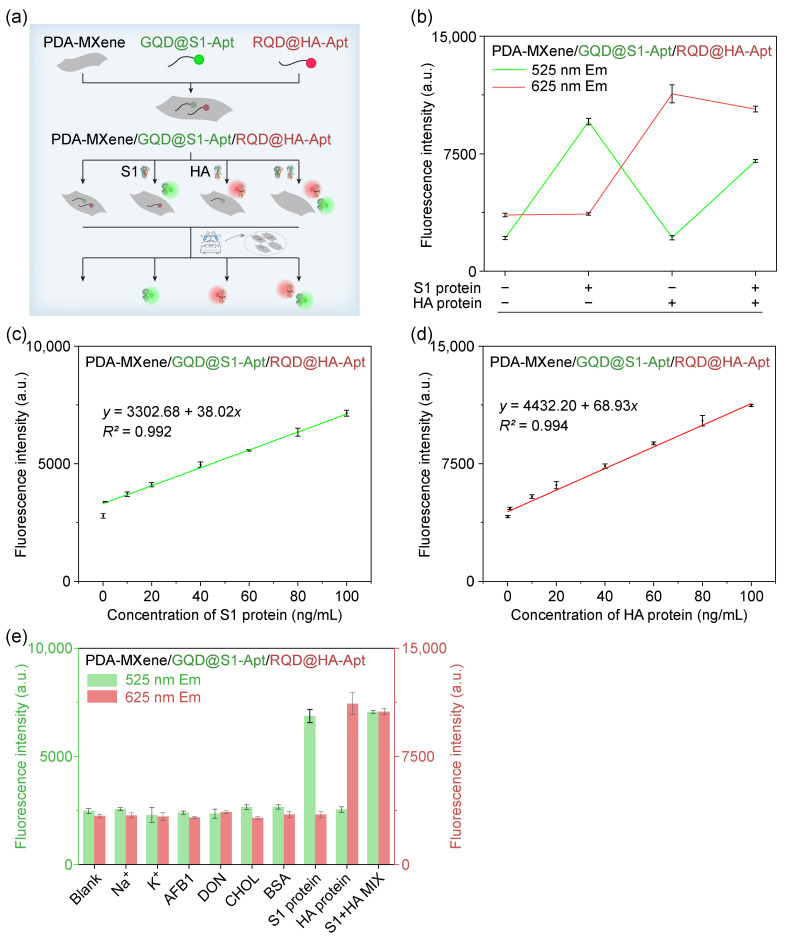
Dual-target simultaneous detection performance. (**a**) Illustration of the PDA-MXene/GQD@S1-Apt/RQD@HA-Apt system for simultaneous detection of S1 and HA proteins. (**b**) Feasibility validation. Fluorescence response signals in the green (525 nm) and red (625 nm) channels under four sample conditions: buffer only (negative control), S1 protein only, HA protein only, and both S1 and HA proteins present. (**c**) Quantification performance of the green channel (GQD@S1-Apt) in response to increasing S1 protein concentrations (0.1–100 ng/mL). Inset: linear calibration curve. (**d**) Quantification performance of the red channel (RQD@HA-Apt) in response to increasing HA concentrations (0.1–100 ng/mL). Inset: linear calibration curve. (**e**) Specificity test of the PDA-MXene/GQD@S1-Apt/RQD@HA-Apt system. The concentration of S1 protein and HA protein was 100 ng/mL, while other interferents were tested at 30 μg/mL. Error bars represent triplicate measurements.

To test the simultaneous detection capability, we integrated the PDA-MXene/GQD@S1-Apt and PDA-MXene/RQD@HA-Apt aptasensors into a single multiplex detection system (Figure 6a). In this dual-target detection system (PDA-MXene/GQD@S1-Apt/RQD@HA-Apt), the emission intensity of the supernatant was measured at 525 nm (GQDs) and 625 nm (RQDs) under an excitation wavelength of 365 nm. As shown in Figure 6b, fluorescence responses were observed at the corresponding emission wavelength when its target was present, while no significant signal was detected in the absence of either target. This suggests that each probe retains its specificity within the dual-target detection system, with no cross-reactivity between the two targets. The absolute fluorescence intensity in the dual-target system was lower than in single-target assays, likely owing to competitive adsorption and steric effects on the PDA-MXene surface. We further tested this dual-target system using S1 and HA proteins at various concentrations (0.1–100 ng/mL) to evaluate its quantitative detection performance. As shown in Figure 6c,d, the fluorescence intensity in both channels increases linearly with target concentration. When detecting S1 protein in the GQD channel, the fitted equation is y = 3302.68 + 38.02x (R^2^ = 0.992), while for HA protein detection in the RQD channel, the relationship followed y = 4432.20 + 68.93x (R^2^ = 0.994). The specificity of the dual-target system was further validated by testing the system with individual or mixed interferent. As shown in Figure 6e, significant fluorescence response was observed only in channels corresponding to their respective target proteins (S1 protein in the green channel, HA protein in the red channel), while all non-target samples showed negligible signals comparable to the blank control. In the mixture containing both S1 and HA (labeled “S1 + HA MIX”), both channels exhibited substantial signal recovery, confirming the presence of S1 protein (green channel based on GQD@S1-Apt) and HA protein (red channel based on RQD@HA-Apt). Each aptamer probe in this dual-target detection system retains its high binding affinity, and no cross-reactivity was detected. The distinct emission wavelengths of the two QD reporters allow clear signal differentiation. Therefore, in an unknown sample, a fluorescence increase at 525 nm alone indicates the presence of S1, an increase at 625 nm alone indicates HA, and increases at both wavelengths indicate the presence of both antigens (co-infection).

The integrated dual-target system enables simultaneous, specific, and quantitative detection of both SARS-CoV-2 S1 protein and H1N1 HA protein within a single assay, supporting the potential of this platform for multiplexed diagnostic applications in complex samples. Because the aptamers are stored as lyophilized powder and rapidly self-assemble with quantum dots upon use, our method offers exceptional long-term storage potential. Lyophilized nucleic acid aptamer powder can be stored long-term at 4 °C or even at room temperature. In contrast, nucleic acid solutions typically require long-term storage at −20 °C or −80 °C, and their stability at 4 °C is significantly reduced. Many previously reported aptasensors require the aptamers to be pre-immobilized on nanomaterials prior to storage; such conjugates often cannot withstand freezing or maintain their activity during storage at 4 °C. In our workflow, we store all key components separately (DBCO-quantum dots, azide-modified aptamer lyophilized powder, PDA-MXene, and filter-sterilized 10× buffer), all at 4 °C. This design provides a practical foundation for long-term storage and serves as a prototype for a dual-target detection kit. When in use, PDA-MXene/quantum dot@Apt composites can be obtained via copper-free click chemistry-mediated self-assembly and adsorption by simply mixing DBCO quantum dots, azide-modified aptamers, and PDA-MXene in sequence. The composite can be purified with a single centrifugation and resuspension using a handheld mini centrifuge to remove excess quantum dots (which are not adsorbed by PDA-MXene). This makes the method suitable for resource-limited environments, and the procedure is simple and straightforward, facilitating standardization.

## 4. Conclusions

This work developed a centrifugation-assisted, aptamer-based fluorescence biosensing method using polydopamine-coated MXene (PDA-MXene) as a carrier of quantum dot-labeled aptamer, which achieves high signal-to-noise detection of respiratory viral antigens. The method demonstrated sensitive and specific single-target detection of SARS-CoV-2 S1 protein (LOD = 0.82 ng/mL) and influenza A HA protein (LOD = 2.11 ng/mL), respectively. In addition, biosensing method allowed the detection of new targets by simply replacing the azide-modified aptamer lyophilized powder. Furthermore, the integration of dual-color quantum dot probes enabled simultaneous detection of both antigens in a single assay with minimal cross-reactivity. Artificial saliva, chosen as real sample, was spiked with the target proteins to investigate the practical applicability of the method, showing good accuracy for both target proteins. This sensing strategy holds promising application for the simultaneous detection of multiple proteins in point-of-care testing and health monitoring fields.

## Figures and Tables

**Table 1:** S1 protein detection in artificial saliva using the PDA-MXene/RQD@S1-Apt system.

Samples	Added (ng/mL)	Determined (ng/mL)	Recovery (%)	RSD (%)
Artificial saliva	0	Nd	/	/
	10	10.10 ± 0.62	100.98	4.58
	20	22.61 ± 1.29	113.05	4.10

**Table 2:** HA protein detection in artificial saliva using the PDA-MXene/RQD@HA-Apt system.

Samples	Added (ng/mL)	Determined (ng/mL)	Recovery (%)	RSD (%)
Artificial saliva	0	Nd	/	/
	10	10.53 ± 1.02	105.32	9.33
	20	20.40 ± 1.99	101.98	8.67

## Data Availability

Data will be made available on request.

## Supplementary Materials

The following supporting information can be downloaded at https://www.mdpi.com/article/10.3390/s26123856/s1, Figure S1: UV-Vis absorption spectrum of MXene; Figure S2: UV-Vis absorption spectrum of dopamine; Figure S3: UV-Vis absorption spectrum of PDA pre-reacted for 3 h; Figure S4: Fluorescence spectra of the free RQD@Apt probe and the mixture of RQD@Apt probe with PDA-MXene; Figure S5: Fluorescence spectra of the free GQD@Apt probe and the mixture of GQD@Apt probe with PDA-MXene; Table S1: Information about aptamers employed in this study.
