# Supplementary material for: Quantum Dot-Based Dual-Fluorescence Aptasensing Platform Using Interface-Engineered MXene for Multiplex Protein Detection
Source: Sensors (Basel). 2026 Jun 17;26(12):3856. doi: 10.3390/s26123856 (PMC13306845; doi:10.3390/s26123856)
Supplement: Supplementary file 1 [file sensors-26-03856-s001.zip › sensors-4337009-supplementary.pdf]

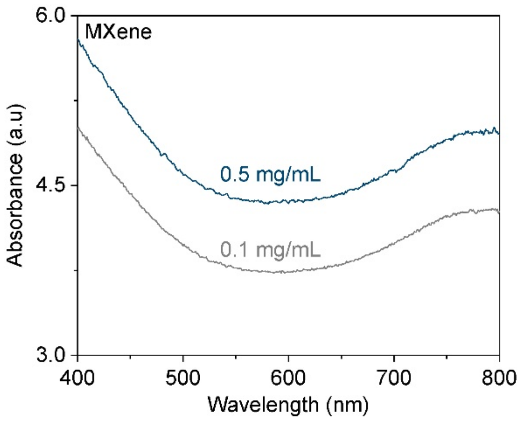

Figure S1. UV-Vis absorption spectrum of MXene.

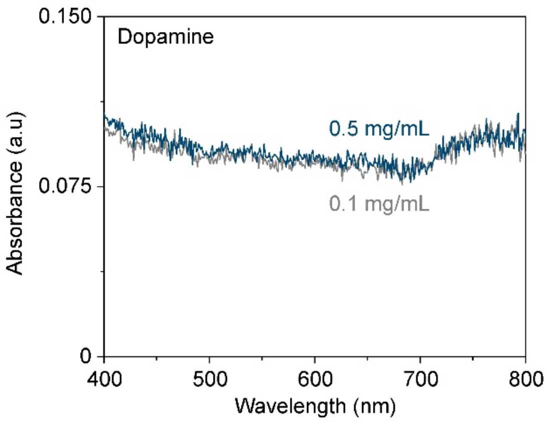

Figure S2. UV-Vis absorption spectrum of dopamine.

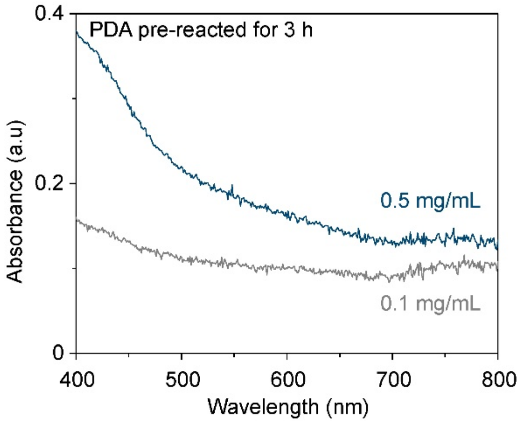

Figure S3. UV-Vis absorption spectrum of PDA pre-reacted for 3 h.

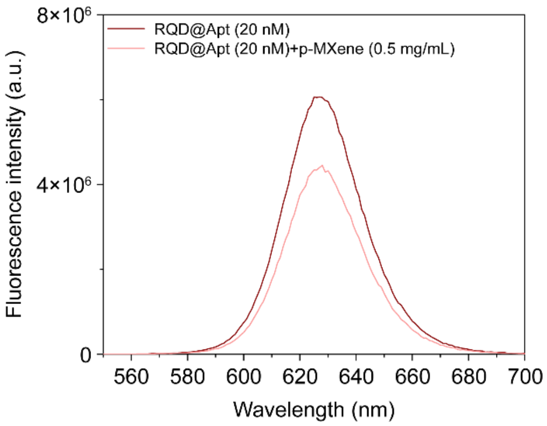

Figure S4. Fluorescence spectra of the free RQD@Apt probe and the mixture of RQD@Apt probe with PDA-MXene.

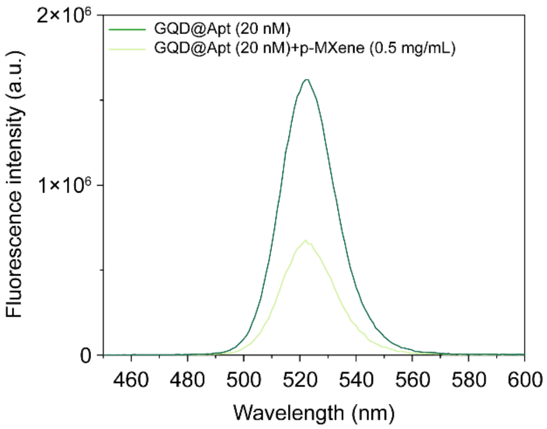

Figure S5. Fluorescence spectra of the free GQD@Apt probe and the mixture of GQD@Apt probe with PDA-MXene.

Table S1. Information about aptamers employed in this study.

Aptamer	Sequence
S1-Apt [1]	5′-N_3-CAG CAC CGA CCT TGC TTT GGG AGT GGC TGG TCC AAG GGC GTT AAT GGA CA-3′
HA-Apt [2]	5′-N_3-GGC AGG AAG ACA AAC AGC CAG CGT GAC AGC GAC GCG TAG GGA CCG GCA TCC GCG GGT GGT CTG TGG TGC TGT-3′

References

1. Li, H.; Zhao, J.; Wu, T.; Fu, Z.; Zhang, W.; Lian, Z.; Cai, S.; Yang, R. Dual ligand-induced photoelectrochemical sensing by integrating Pt/MoS2 heterostructure and Au polyhedra for sensitive detection of SARS-CoV-2. *Sensors and Actuators B: Chemical* **2023**, *376*, 132970. doi:10.1016/j.snb.2022.132970.
2. Tseng, Y.-T.; Wang, C.-H.; Chang, C.-P.; Lee, G.-B. Integrated microfluidic system for rapid detection of influenza H1N1 virus using a sandwich-based aptamer assay. *Biosensors and Bioelectronics* **2016**, *82*, 105-111. doi:10.1016/j.bios.2016.03.073.
